# Uncovering New Biomarkers for Prostate Cancer Through Proteomic and Network Analysis

**DOI:** 10.3390/biology14030256

**Published:** 2025-03-04

**Authors:** Rossana Rossi, Elena Monica Borroni, Ishak Yusuf, Andrea Lomagno, Mohamed A. A. A. Hegazi, Pietro Luigi Mauri, Fabio Grizzi, Gianluigi Taverna, Dario Di Silvestre

**Affiliations:** 1 Institute for Biomedical Technologies—National Research Council, 20054 Segrate, Milan, Italy; rossana.rossi@itb.cnr.it (R.R.); ishak.yusuf@itb.cnr.it (I.Y.); andrea.lomagno@itb.cnr.it (A.L.); pierluigi.mauri@itb.cnr.it (P.L.M.); 2 Department of Medical Biotechnologies and Translational Medicine, University of Milan, 20054 Segrate, Milan, Italy; elena.borroni@unimi.it; 3 Department of Immunology and Inflammation, IRCCS Humanitas Research Hospital, 20089 Rozzano, Milan, Italy; lunamohamed75@gmail.com (M.A.A.A.H.); fabio.grizzi@humanitasresearch.it (F.G.); 4 Department of Biomedical Sciences, Humanitas University, 20072 Pieve Emanuele, Milan, Italy; 5 Department of Urology, Humanitas Mater Domini, 21100 Castellanza, Varese, Italy; gianluigi.taverna@humanitas.it

**Keywords:** prostate cancer, urine, proteomics, network analysis, hubs, TCGA

## Abstract

Background: Prostate cancer (PCa), is the second most prevalent solid tumor among men worldwide (7.3%), and the leading non-skin cancer in USA where it represents 14.9% of all new cancer cases diagnosed in 2024. This multifactorial disease exhibits substantial variation in incidence and mortality across different ethnic groups and geographic regions. Although prostate-specific antigen (PSA) remains widely used as a biomarker for PCa, its limitations reduce its effectiveness for accurate detection. Consequently, finding molecules that can either complement PSA and other biomarkers is a major goal in PCa research. Methods: Urine samples were collected from healthy donors (*n* = 5) and patients with low- and high-risk PCa (4 and 7 subjects, respectively) and were analyzed using proteomic data-derived systems and biology approaches. The most promising proteins were further investigated by means of The Cancer Genome Atlas (TCGA) database to assess their associations with clinical and histopathological characteristics in a larger in silico patient population. Results: By evaluating the variations in the urinary proteome as a mirror of the changes occurring in prostate tumor tissue, components of complement and coagulation cascades and glutathione metabolism emerged as hallmarks of low- and high-risk PCa patients, respectively. Moreover, our integrated approach highlighted new potential biomarkers, including CPM, KRT8, ITIH2, and RCN1. Conclusions: The good overlap of our results with what is already reported in the literature supports the new findings in the perspective of improving the knowledge on PCa. Furthermore, they increase the panel of biomarkers that could enhance PCa management. Of course, further investigations on larger patient cohorts are required.

## Simple Summary

Prostate-specific antigen (PSA) remains widely used as a biomarker for prostate cancer (PCa). However, due to the heterogeneity of this disease, its use has some limitations for accurate diagnosis. The main objectives of our study include the identification of proteins that can complement the already available biomarkers, and the evaluation of urinary proteome changes as a mirror of changes occurring in prostate cancer tissue. Using a proteomic data-derived systems biology approach, we found some components of complement and coagulation cascades and glutathione metabolism as hallmarks of low- and high-risk PCa patients, respectively. Furthermore, we selected a set of promising biomarkers, including CPM, KRT8, ITIH2, and RCN, that could improve the management of PCa.

## 1. Introduction

Prostate cancer (PCa) is one of the most commonly diagnosed cancers worldwide, accounting for 7.3% of all cancer cases [1]. It is the most diagnosed cancer and the second leading cause of cancer-related deaths among men in the U.S. [2]. Here, in 2024, it is estimated that there will be 299,010 new cases, representing 14.9% of all new cancer cases. Additionally, an estimated 35,250 deaths will occur, accounting for 5.8% of all cancer deaths [3]. While many prostate tumors are highly aggressive and eventually lead to death, many more are indolent and never metastasize. This heterogeneity poses challenges in screening and treatment of PCa [2].The initial evaluation typically begins with a digital rectal examination (DRE) and a prostate-specific antigen (PSA) test [4]. Following this, around 12 tissue samples are generally collected from the prostate gland via a biopsy, either using transrectal ultrasound (TRUS) guidance or MRI-targeted fusion techniques. A definitive diagnosis of PCa is made by microscopic examination of these samples, with the assignment of a Gleason score (GS) [5], as recommended by current medical guidelines. After diagnosis, imaging of the pelvic region or entire body may be performed using multi-parametric MRI (mpMRI), computed tomography (CT), or positron emission tomography (PET). Risk stratification is then applied using established algorithms like D’Amico, International Society of Urological Pathology (ISUP), National Comprehensive Cancer Network (NCCN), and Cancer of the Prostate Risk Assessment (CAPRA) [6]. PSA, a protein produced by the prostate gland, is commonly used as a marker for prostate health, with its levels measured by immunoassay. Elevated PSA levels may indicate conditions such as benign prostatic hyperplasia or PCa. Prostate biopsy remains the gold standard for confirming a prostate cancer diagnosis, though it is invasive and carries a post-biopsy sepsis risk of up to 2.4%. Furthermore, the PROMIS trial found that this method underestimated clinically significant PCa, defined as a Gleason score of ≥4 + 3 or a maximum cancer core length of 6 mm or more, in 18% of cases [7].

Like many types of cancer, prostate cancer is asymptomatic in its early stages. It is characterized by various clinical, pathological, and molecular features, necessitating the development of personalized and integrated molecular approaches for improved diagnosis, treatment, and prognosis [8,9,10,11,12]. To achieve this goal, new diagnostic strategies are evaluating the use of mRNA [13] and miRNA [14] biomarkers, as well as the combination of MRI and artificial intelligence (AI) systems [15]. Moreover, modern proteomics is facilitating the shift from relying on a single marker, such as PSA, to utilizing panels of biomarkers. This transition aims to enable earlier and more accurate diagnosis [16]. Biomarker discovery has been conducted through the analysis of biological fluids like serum [12,17], plasma [17,18] and urine [19,20], as well as by characterizing the proteome of tissue biopsies [21,22]. Additionally, the analysis of extracellular vesicles [23,24], including exosomes [25,26], is increasingly being adopted for this purpose.

Although most studies aim to identify biomarkers for early diagnosis, some of them have also turned their attention to shedding light on the mechanisms underlying tumor development [27,28], while others have sought to correlate biomarker profiles with tumor aggressiveness [29,30]. However, few studies have considered the reconstruction of protein–protein interaction (PPI) network models, which reveal the relationships between proteins [31,32]. In the present study, we analyzed urine samples from healthy donors (HDs) and PCa patients at the proteomics level, categorizing them based on clinical Gleason scores into low-risk (LRPCa) and high-risk PCa (HRPCa) groups. In addition to identifying potential biomarkers linked to disease stages, we used the identified proteins to reconstruct PPI network models for each group [33]. These models were analyzed both functionally, to identify PPI modules, and topologically, to select key regulatory proteins, or “hubs” and “bottlenecks”, that play critical roles in the underlying biological systems [34].

## 2. Materials and Methods

### 2.1. Sample Collection and Isolation of Total Proteins from Urine

A total of 16 participants were included in this study. Urine samples were collected from five HD with a mean age of 29 ± 3 years, four individuals with LRPCa with a mean age of 66 ± 10 years and GS < 7, and seven individuals with HRPCa with a mean age of 64 ± 9 years and GS > 7. Total proteins were isolated from 1 mL of urine using the Urine Protein Concentration Micro Kit (Cat# 17400) (Norgen Biotek, Thorold, ON, Canada). The isolation was performed according to the manufacturer’s instructions.

### 2.2. Protein Extraction and Enzymatic Digestion

Two hundred microliters per protein sample, collected at the end of isolation, were concentrated to 50 μL in a vacuum concentrator at 60 °C and treated with RapiGestTMSF reagent (Waters Co., Milford, MA, USA) at the final concentration of 0.25% (*w*/*v*). The resulting suspensions were incubated under stirring at 100 °C for 20 min. Subsequently, the samples were cooled to room temperature and centrifuged 10 min at 2200× *g*. The protein concentration was assayed using the Invitrogen Qubit Protein BR Assay Kit (Life Technologies Corporation, Thermo Fisher, Eugene, OR, USA) and 50 μg proteins from each sample were digested overnight at 37 °C by adding Sequencing-grade Modified Trypsin (Promega Inc., Madison, WI, USA) at an enzyme/substrate ratio of 1:50 (*w*/*w*) in 0.1 M ${\mathrm{NH}}_{4}$${\mathrm{HCO}}_{3}$ pH 7.9 buffer with 10% CH_3_CN. The next day, an additional aliquot of trypsin (1:100 *w*/*w*) was added. After 4h, the enzymatic digestion was stopped by acidification with 0.5% Trifluoroacetic Acid (TFA) (Sigma-Aldrich Inc., St. Louis, MO, USA), and a subsequent incubation at 37 °C for 45 min completed the RapiGest acid hydrolysis. Water immiscible degradation products were removed by centrifugation at 13,000 rpm for 10 min. Finally, the obtained mixtures were desalted through PierceTM C-18 spin columns (Thermo Fisher Scientific, Pierce Biotechnology, Rockford, IL, USA) and were resuspended in 0.1% formic acid (Sigma-Aldrich Inc., St. Louis, MO, USA) in water (LC-MS Ultra CHROMASOLV™, Honeywell Riedel-de HaenTM, Muskegon, MI, USA) at a concentration of 0.1 μg/μL.

### 2.3. Proteomic Analysis by nanoLC-MS/MS

Proteomic analysis was performed through the LTQ-Orbitrap XL-ETD mass spectrometer (Thermo Fisher Scientific, San José, CA, USA) coupled with the Eksigent nanoLC-Ultra^®^ 2D System (Eksigent, part of AB SCIEX Dublin, CA, USA) configured in trap-elute mode. Briefly, samples (0.8 μg injected) were first loaded on a trap (200 μm × 500 μm ChromXP C18-CL, 3 μm, 120 Å) and washed with the loading pump running in isocratic mode with 0.1% formic acid in water for 10 min at a flow of 3 μL/min. The trapped mixture was then eluted on a nano reversed-phase column (75 μm × 15 cm ChromXP C18-CL, 3 μm, 120 Å) using a 130 min gradient of eluent B (eluent A, 0.1% formic acid in water; eluent B, 0.1% formic acid in acetonitrile) at a flow rate of 300 nL/min. Particularly, the gradient was: from 5–10% B in 5 min, 10–40% B in 85 min, 40–95% B in 27 min and holding at 95% B for 10 min. The eluted peptides were finally ionized through a nanoelectrospray ion source (Thermo Fisher Scientific) and analyzed by a LTQ-Orbitrap XL-ETD; each sample was analyzed in at least 3 technical replicates. The spray capillary voltage was set at 1.7 kV and the ion transfer capillary temperature was held at 220 °C. Further analytical details may be found in Bari et al. [35].

### 2.4. MS/MS Data Processing

The Raw data collected following the analysis by LTQ-Orbitrap XL-ETD were processed by SEQUEST HT algorithm contained in the Proteome Discoverer 2.5 software (Thermo Fisher Scientific, San José, CA, USA). The experimental MS/MS spectra were compared against the theoretical mass spectra reconstructed by in silico digestion of *Homo sapiens* protein sequences downloaded from UNIPROT (www.uniprot.org) in December 2023. The following parameters of searching were set: trypsin enzyme, 3 missed cleavages per peptide, mass tolerances on precursor ions were set to ±100 ppm, while ±0.8 Da were set for fragment ions. The percolator node was used with a target-decoy strategy to give final false discovery rates (FDR) ≤ 0.01 (strict) based on q-values, considering a maximum deltaCN of 0.05. Only peptides with a minimum peptide length of six amino acids, confidence at “Medium” level and rank 1 were considered. Protein grouping and strict parsimony principle were applied.

### 2.5. Enrichment Analysis

The functional evaluation of the characterized protein profiles was performed by the functional annotation tool included in the STRING database [36]. For each subject, the enriched GO terms (Biological Process (BP), Molecular Function (MF), Cellular Component (CC) and COMPARTMENTS) and Pathways (KEGG, Reactome and WikiPathways) were extracted (FDR ≤ 0.05). These functional modules were compared by linear discriminant analysis (LDA) and those with F ratio ≥ 3.5 and *p*-value ≤ 0.05 were selected as differentially enriched among HD, LRPCa and HRPCa groups. Finally, they were represented by tree charts built using the ggtree and ggplot2 R package (v3.5.1) [37] and Circos tool (v0.52) [38].

### 2.6. Label-Free Quantitative Analysis

The characterized protein profiles were semi-quantitatively compared by a label-free approach, as previously reported [34]. Specifically, the Peptide Spectrum Matches (PSMs) values were normalized using a total signal normalization method [39]. The average PSMs per group were processed by Spearman’s correlation. Data matrix dimensionality (16 subjects and 2490 proteins) was reduced by LDA and proteins with F ratio ≥ 3.5 and *p*-value ≤ 0.05 were selected as differentially abundant (DAPs). To recover protein not selected by LDA, due to the subjectivity that can lead to high within-group variation, we performed a further DAPs extraction by taking into account the protein Identification Frequency (IF); in this case, we retained as DAPs those with IF > 50% in one group, and IF < 50% in at least one of the others. Finally, pairwise comparisons (HD vs. LRPCa; HD vs. HRPCaλ and LRPCa vs. HRPCa) were evaluated by Differential Average (DAve) index:

$$({PSMs}_{A}-{PSMs}_{B})/({PSMs}_{A}+{PSMs}_{B})/0.5$$

where A and B represent the conditions compared); conventionally, the DAve index of proteins exclusively identified only in one of the conditions under comparison was set to ±2. Specifically, positive DAve values indicate proteins up-regulated in A (and down-regulated in B), while negative DAve values indicate proteins up-regulated in B (and down-regulated in A); a −0.4 ≥ DAve ≥ 0.4 was considered [40]. Selected DAPs were further processed by Hierarchical Clustering and Principal Component Analysis (PCA) to graphically check the homogeneity of expression levels in subjects belonging to the same group and exclude the presence of outliers. All data processing was performed using JMP 15.2 SAS software.

### 2.7. Reconstruction of PPI Network Model and Functional Modules Identification

A PPI network model was reconstructed by STRING Cytoscape’s APP [36] starting from DAPs selected by IF and LDA approaches (*n* = 141); only protein–protein interactions “databases” and/or “experiments” were annotated, with scores ≥ 0.3 and ≥0.15 retained. The proteins were grouped in PPI functional modules by the support of STRING Cytoscape’s APP and BINGO 2.44 [41]; as for BINGO 2.44, *Homo sapiens* organism, hypergeometric test and Benjamini–Hochberg FDR correction (≤0.01) were set.

### 2.8. Topological Analysis of PPI and Co-Expression Network Models

A PPI network per group was reconstructed starting from proteins, respectively, found in at least 3 up to 5 HD subjects, at least 2 up to 4 LRPCa subjects and at least 4 up to 7 HRPCa subjects. The HD, LRPCa and HRPCa PPI models thus reconstructed were topologically analyzed by Centiscape Cytoscape’s APP [42], as previously reported [43]. Diameter, Average Distance, Degree, Betweenness, Centroid, Stress, EigenVector, Bridging, Eccentricity, Closeness, Radiality and Edge centralities were calculated. Betweenness coupled with Centroid and Betweenness coupled with Bridging were used to select hubs and bottlenecks, respectively; in particular, nodes with both values above the average were retained [33]. Statistical significance of topological results was tested by randomized network models [44]; *n* = 1000 random models per group were reconstructed and analyzed by *in house* R scripts based on VertexSort (to build random models), igraph (to compute centralities), and ggplot2 (to plot results) libraries.

### 2.9. TCGA Bioinformatic Analysis

To investigate the correlation between proteomic findings and clinicopathological characteristics, we analyzed proteins predominantly abundant in urine samples from HD, or in patients with a PCa diagnosis, using data from The Cancer Genome Atlas (TCGA) and the UALCAN public library [45]. Similarly, we examined proteins characterizing the urine of LRPCa and HRPCa individuals. This two-step approach allowed us to evaluate the association between protein profile and disease severity, helping us identify potential biomarkers linked to clinical risk. The Tumor Immune Estimation Score TIMER 2.0 database was instead used to analyze the association between predominantly expressed genes and immune infiltration [46].

## 3. Results

A cohort of 16 subjects, including five HD, four subjects with LRPCa and seven subjects with HRPCa, was investigated.

### 3.1. Protein Profiling of Urine from Healthy Controls and Patients Affected by Prostate Cancer at Low- and High-Risk Level

**Figure 1:**
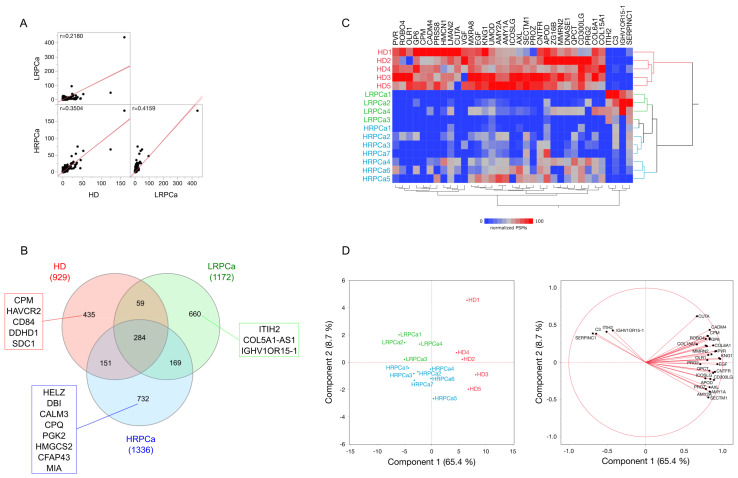
Proteomic analysis was performed on urine samples from HD and PCa patients categorized as low- (LRPCa) and high-risk (HRPCa). (**A**) Spearman’s correlation was used to compare the global protein profiles of HD, LRPCa, and HRPCa groups. (**B**) A Venn diagram shows proteins uniquely identified in HD, LRPCa, and HRPCa groups. Red, green, and blue rectangles show proteins found in at least 40% of subjects per group. (**C**) Hierarchical clustering and (**D**) Principal Component Analysis (PCA) were conducted using peptide spectrum matches (PSMs) of differentially abundant proteins (DAPs) extracted via Linear Discriminant Analysis (LDA); both show a good grouping based on the health status of the subjects considered. Principal Component1 (PC1) and Principal Component2 (PC2) accounted for 65.4% and 8.7% of the variance, respectively. Hierarchical clustering employed *Euclidean* distance metrics and *Ward*’s method.

After performing 48 nano-liquid chromatography-mass spectrometry/high resolution mass spectrometry (nLC-hrMS/MS) runs of urine samples from PCa patients and HD, 2490 distinct proteins were identified, each confirmed by at least one unique peptide. The complete protein profile of each subject is provided in Table S1. A comparison of the average protein profiles for HD, LRPCa, and HRPCa was conducted using *Spearman’s* correlation (Figure 1A). The HD proteome exhibited a low correlation with HRPCa (r = 0.35) and an even lower correlation with LRPCa (r = 0.22). As expected, LRPCa and HRPCa had the highest correlation (r = 0.42), reflecting the similarities between the two PCa groups. These differences between the groups were further emphasized in a Venn diagram, which shows the number of shared and group-specific proteins (Figure 1B). In detail, 284 proteins were common across all groups, while 435, 660, and 732 proteins were unique to HD, LRPCa, and HRPCa, respectively. Notably, HD shared fewer proteins with LRPCa (*n* = 59) than with HRPCa (*n* = 151), while 169 proteins were shared between LRPCa and HRPCa.

### 3.2. Differentially Abundant Proteins (DAPs) by Comparing Urine Protein Profiles from Healthy Controls and Patients Affected by Prostate Cancer at Low- and High-Risk Level

A first comparison of HD, LRPCa, and HRPCa profiles was done by a Venn diagram and by taking into account the identification frequency (IF) of group-specific proteins. Among the proteins that characterize the urinary profiles of HD with greater IF (≥40%), we found Carboxypeptidase M (CPM), Hepatitis A virus cellular receptor 2 (HAVCR2), SLAM family member 5 (CD84), Phospholipase DDHD1 (DDHD1) and Syndecan-1 (SDC1). Similarly, Inter-Alpha-Trypsin Inhibitor Heavy Chain 2 (ITIH2), Putative uncharacterized protein encoded by COL5A1-AS1 (COL5A1-AS1) and Immunoglobulin heavy variable 1/OR15-1 (IGHV1OR15-1) were found in LRPCa subjects. While, Helicase With Zinc Finger (HELZ), Acyl-CoA-binding protein (DBI), Calmodulin-3 (CALM3), Carboxypeptidase Q (CPQ), Phosphoglycerate kinase 2 (PGK2), Hydroxymethylglutaryl-CoA synthase (HMGCS2), Cilia- and flagella-associated protein 43 (CFAP43) and Melanoma-derived growth regulatory protein (MIA) were exclusively identified in HRPCa (Figure 1B). In this scenario, CPM, ITIH2 and HELZ were the proteins identified with the highest frequency in HD (IF = 100%), LRPCa (IF = 100%) and HRPCa (IF = 57%), respectively. However, following this approach, other potential biomarkers emerged for HRPCa. This is the case of Cofilin-1 (CFL1), being present in almost all HRPCa cases (IF = 86%), while found in only 25% of LRPCa cases and absent in HD (Table S2). Of note, ITIH2 has already been suggested as a potential urinary biomarker for PCa [47], and Hydroxymethylglutaryl-CoA synthase (HMGCS2) has been observed to be significantly elevated at both the transcript and protein levels in high-grade PCa human tissues [48,49].

Following the evaluation of IF, we selected a total of 56 DAPs to which we added other 85 identified by processing PSMs through LDA (*p* ≤ 0.05) and DAve index [34,50]; as a result, we collected a total of 141 DAPs (Table S2). Hierarchical clustering and PCA were applied to verify that all subjects were correctly grouped according to their clinical classification. Interestingly, two main branches emerged: one consisting of HD and the other comprising PCa patients (Figure 1C). This grouping was further supported by PCA (Figure 1D), where Principal Component1 (PC1), accounting for 65.4% of the variance, distinguished HD from PCa patients, while Principal Component2 (PC2) primarily separated LRPCa from HRPCa patients.

In addition to ITIH2, LRPCa patients showed a higher abundance of Serpin Family C Member 1 (SERPINC1), Complement C3 (C3), and Immunoglobulin Heavy Variable 1/OR15-1 (IGHV1OR15-1), with SERPINC1 [51] and Complement C3 [52] previously associated with PCa. In contrast, apart from HELZ, which was detected in four out of seven HRPCa patients (and absent in HD and LRPCa), no other proteins were uniquely abundant in HRPCa subjects. Rather than the presence of novel protein biomarkers, both LRPCa and HRPCa patients showed a reduction in some proteins (e.g., PVR, MXRA8, CPM, and EGF) characterizing HD urine. Notably, reduced urinary EGF levels have been associated with various kidney disorders [53]. CPM and Pro-epidermal growth factor (EGF) have been linked in urine, with CPM serving as the major EGF-metabolizing enzyme in these fluids [54].

### 3.3. Functional Modules Marking the Urine Proteome of Healthy Controls and Patients Affected by Prostate Cancer at Low- and High-Risk Levels

**Figure 2:**
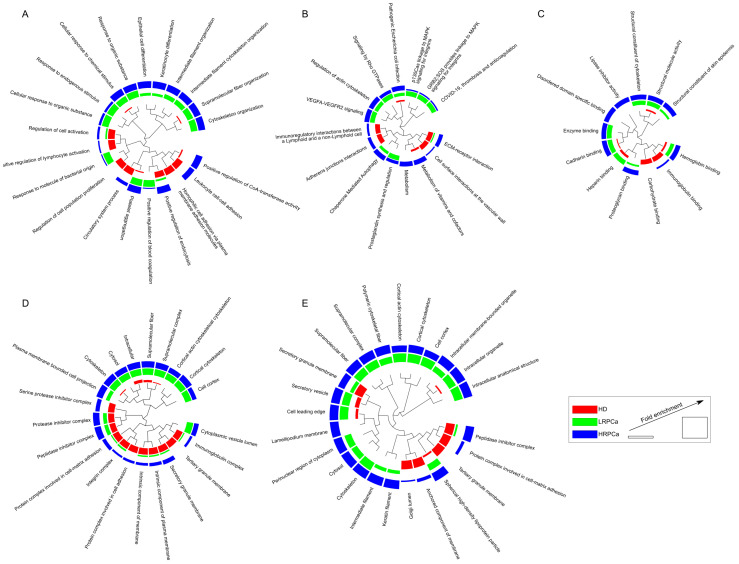
Functional enrichment analysis. (**A**) GO biological process, (**B**) pathways, (**C**) GO molecular function, (**D**) COMPARTMENT, (**E**) and GO cellular component differentially enriched by comparing HD (red), LRPCa (green) and HRPCa (blue) groups. The functional modules were enriched starting from the whole protein profile characterized per subject (FDR ≤ 0.05), while the enrichment profiles were compared by linear discriminant analysis (LDA, *p* ≤ 0.05).

To gain further insights into the biological roles of the protein profiles from the urine of HD, LRPCa and HRPCa, we conducted an enrichment analysis based on GO Biological Process, GO Molecular Function, GO Cellular Component, Compartment, and Pathways (Figure 2 and Table S3). This analysis revealed an enrichment in keratinocyte and epithelial differentiation processes in both LRPCa and HRPCa groups. Additionally, it highlighted biological processes related to cytoskeleton organization, supramolecular fibers, and intermediate filaments. In contrast, processes such as the regulation of lymphocyte activation and cell population proliferation were predominantly enriched in the HD group (Figure 2A).

The correlation between the immune system and HD was reinforced by the enrichment of the “Immunoregulatory interactions between a Lymphoid and a non-Lymphoid cell” pathway (Figure 2B). Consistently, proteins with immunoglobulin-binding functions were enriched in HD, while HRPCa showed an enrichment of lipase inhibitor activity and proteoglycan-binding functions (Figure 2C). Pathway analysis further highlighted the significance of cytoskeleton organization, angiogenesis (VEGFA-VEGFR2 signaling), and apoptosis (Signaling by Rho GTPases) in both LRPCa and HRPCa patients (Figure 2B). These functional insights were also reflected in the subcellular localizations most represented (Figure 2D,E). While structural proteins were more prevalent in LRPCa and HRPCa, the immunoglobulin complex and tertiary granule membrane were notably enriched in HD. Interestingly, protease and peptidase inhibitors displayed a similar trend in both HD and HRPCa subjects, but were significantly less enriched in LRPCa subjects; a trend extended to other proteins and functional modules as well.

**Figure 3:**
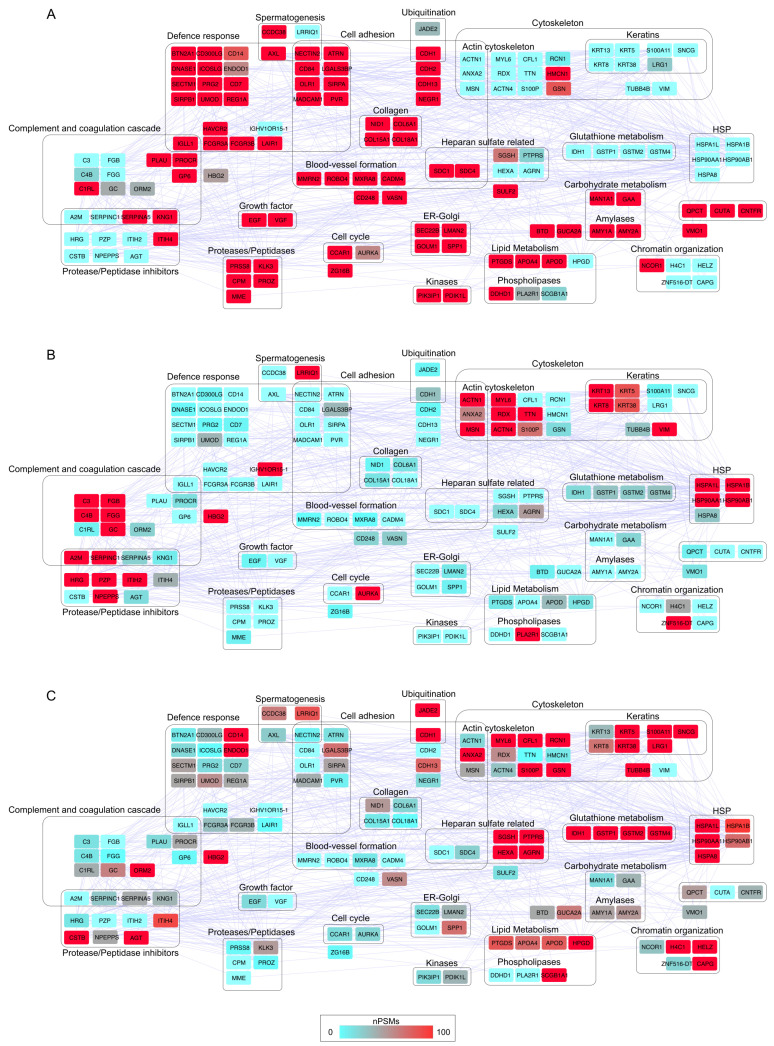
Protein–protein interaction (PPI) network and functional modules differentially abundant in (**A**) HD, (**B**) LRPCa and (**C**) HRPCa. The PPI network was reconstructed starting from proteins selected as differentially abundant (DAPs, *n* = 141); only database (score ≥ 0.3) and experiment (score ≥ 0.15) annotated interactions were considered. The functional modules were defined through STRING and BINGO Cytoscape’s APPs (*p* ≤ 0.05). The color code, from blue to red, is based on the normalized PSM values (range 0–100) and indicates low (blue) and high (red) abundant proteins.

To further investigate the functional groups characterizing the urine of HD and PCa patients, the differentially abundant proteins (DAPs) were classified into 21 protein–protein interaction (PPI) functional modules (Figure 3). Globally, the analysis revealed a reduced release of proteins in PCa patients that were abundant in HD, a trend that was evident at the system level as well. HD urine contained higher concentrations of consistent modules involved in defense response, cell adhesion, and proteolysis. In addition to smaller modules associated with carbohydrate and lipid metabolism, HD also displayed proteins potentially involved in the blood-vessel formation, including ROBO4 [55] and CADM4 [56], which have an inhibitory function. On the other hand, LRPCa patients exhibited higher levels of proteins involved in the complement and coagulation cascade [51,57], keratins, actin cytoskeleton-related proteins and heat shock proteins (HSPs) [58]. These modules were even more abundant in HRPCa patients, alongside proteins related to heparan sulfate metabolism, chromatin organization, and glutathione metabolism. Interestingly, HRPCa patients showed complement and coagulation cascade levels similar to HD, rather than LRPCa, indicating a distinct protein profile.

### 3.4. Network Hubs and Bottlenecks in Urine of Healthy Controls and Patients Affected by Prostate Cancer at Low- and High-Risk Levels

**Figure 4:**
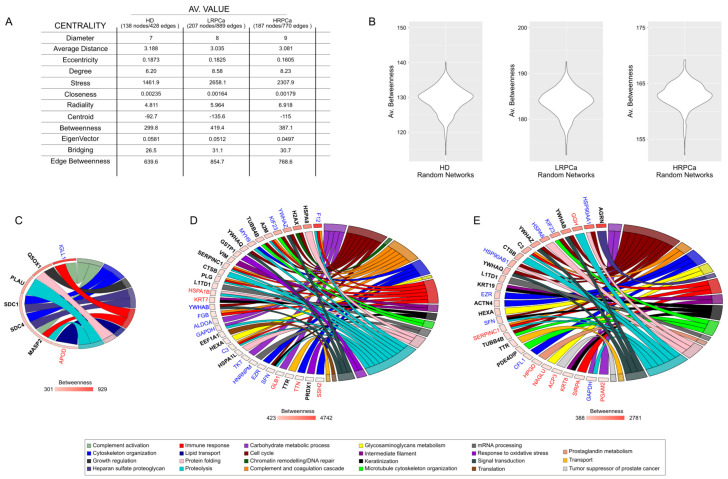
Network topology. (**A**) Media netw (**A**) Average network centralities calculated for the HD, LRPCa and HRPCa PPI network models. (**B**) Violin plots of betweenness from HD, LRPCa and HRPCa PPI random network models; the average value of betweenness in real models (**A**) differs from that of random networks validating the selection of hubs/bottlenecks. (**C**) Hubs and bottlenecks from HD, (**D**) LRPCa and (**E**) HRPCa; specifically, blue gene names indicate hubs selected by betweenness and centroid, red gene names indicate bottlenecks selected by betweenness and bridging, while black bold gene names indicate hubs/bottlenecks selected by betweenness, centroid and bridging centralities. Hubs and bottlenecks were related to their main functions, and the GO chord plots show that in PCa patients, they mainly fall in cell cycle, complement and coagulation cascade, immune response and proteolysis.

To better understand the mechanisms behind the presence of proteins in the urine of HD and PCa patients, we reconstructed and topologically analyzed a PPI network model for each group. The LRPCa and HRPCa networks exhibited high diameters and low average distances. Combined with a higher average degree, it suggests an increased ability for proteins to communicate and a greater tendency to form functional modules (Figure 4A). In this context, hubs and bottlenecks represent key regulatory proteins that could play a major role in disease progression. After validating the networks through random models (Figure 4B), we identified 7 hubs/bottlenecks for HD, 34 for LRPCa, and 29 for HRPCa (Figure 4C–E). Of note, 15 hubs/bottlenecks were in common between LRP and HRP (Table S4). Several of these proteins have previously been linked to PCa. Notably, components of the 14-3-3 protein family, including 14-3-3 protein theta (YWHAQ) [59], 14-3-3 protein beta/alpha (YWHAB) [60] and 14-3-3 protein sigma (SFN), were present, along with inflammation-related proteins such as Complement C3 (C3) and Antithrombin-III (SERPINC1) [51]. Microtubule-related proteins like Kinesin-like protein KIF23 (KIF23) [61] and Tubulin beta-4B chain (TUBB4B) [51] were also highlighted.

Heat shock proteins, particularly HSPA8 (found in both patient groups), as well as HSPA1B and HSPA1L, were found in LRPCa, reflecting the role of the HSP70 family in tumor cell survival [62]. Conversely, HRP networks were characterized by the presence of Heat shock protein HSP90-alpha (HSP90AA1) and HSP90-beta (HSP90AB1) [63], which are also important for cancer progression. In HRPCa, additional notable hubs included Agrin (AGRN) [43,64,65], Keratin, type I cytoskeletal 19 (KRT19) [66] and Keratin type II cytoskeletal 8 (KRT8) [67]. Among HD hubs/bottlenecks, two heparan sulfate proteoglycans, such as Syndecan-1 (SDC1) and Syndecan-4 (SDC4), emerged. They have been inversely associated with PCa aggressiveness [68]. Overall, LRPCa hubs/bottlenecks were enriched in functional categories such as cell cycle regulation, complement and coagulation cascades, immune system processes, and proteolysis. While these categories were also represented in HRPCa, there was a stronger focus on cell cycle processes, keratinization, and microtubule cytoskeleton organization.

### 3.5. TCGA Bioinformatic Analysis: HD vs. PCa

To investigate the correlation between proteomic findings and clinico-pathological characteristics, we analyzed proteins predominantly expressed in urine samples from HD compared to those with a PCa diagnosis, using data from TCGA and the UALCAN public library (https://ualcan.path.uab.edu/), accessed on 15 November 2024.

**Figure 5:**
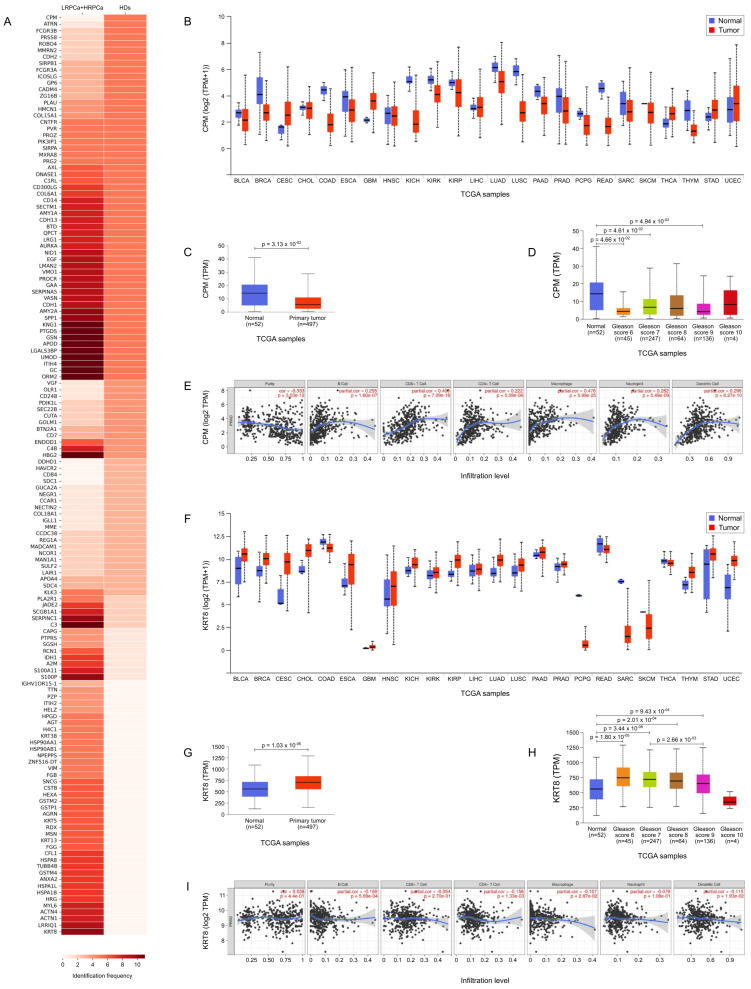
TCGA bioinformatic analysis. (**A**) Heatmap illustrating the frequency of protein expression in urine samples from healthy donors (HD) and prostate cancer-affected (PCa) patients. (**B**) TCGA expression data for Carboxypeptidase M (CPM) across a pan-cancer dataset, comparing expression levels in tumor tissues (red) versus their normal counterparts (blue). (**C**) CPM expression in normal vs. primary tumor. (**D**) CPM expression in normal versus PCa tissues with different Gleason score (GS). (**E**) TIMER analysis correlating CPM expression with immune cell infiltration. (**F**) TCGA expression data for Keratin, type II cytoskeletal 8 (KRT8) across a pan-cancer dataset, comparing expression levels in tumor tissues (red) versus their normal counterparts (blue); Wilcoxon test *p* ≤ 0.05. (**G**) KRT8 expression in normal vs. primary tumor. (**H**) KRT8 expression in normal versus PCa tissues with different Gleason score (GS); Wilcoxon test *p* ≤ 0.05. (**I**) TIMER analysis correlating KRT8 expression with immune cell infiltration.

Figure 5A highlights the most prominently expressed and statistically significant proteins identified in urine samples from HD compared to those from individuals diagnosed with PCa. Notably, Carboxypeptidase M (CPM) was detected in all urine samples from HD (5 out of 5) and was entirely absent in samples from PCa patients (*p* = 0.00063, Table 1). In contrast, Keratin type II cytoskeletal 8 (KRT8) was predominantly found in 91% (10 out of 11) of urine samples from PCa patients and was not detected in those from HD (*p* = 0.0034).

Figure 5B,F illustrate the TCGA expression data for CPM and KRT8, respectively, across a pan-cancer dataset, comparing expression levels in tumor tissues versus their normal counterparts. This analysis highlights distinct expression patterns for CPM and KRT8, suggesting their potential roles as biomarkers across various cancers. In PCa patients specifically, expression differences for both proteins were statistically significant (*p* = 3.13 $\times \;10^{-2}$ for CPM and *p* = 1.03 $\times \;10^{-6}$ for KRT8, shown in Figure 5C and Figure 5G, respectively). Further analysis of CPM expression in normal versus PCa tissues revealed statistically significant differences across several GS, including GS 6 (*p* = 4.66 $\times \;10^{-2}$), GS 7 (*p* = 4.61 $\times \;10^{-2}$), and GS 9 (*p* = 4.94 $\times \;10^{-2}$) (Figure 5D). When examining CPM expression by nodal status, a significant difference was found between normal tissues and N0 status (*p* = 4.49 $\times \;10^{-2}$), though no significant differences were observed between normal and N1 or N0 and N1. Additionally, TIMER analysis indicated that CPM expression is correlated with immune cell infiltration, specifically with B cells, CD4+ T cells, CD8+ T cells, macrophages, neutrophils, and dendritic cells (Figure 5E), suggesting a potential role for CPM in the tumor microenvironment and immune response. Also KRT8 expression in normal versus PCa tissues revealed statistically significant differences across GS, including normal prostate tissue versus GS 6 (*p* = 1.80 $\times \;10^{-5}$), GS 7 (*p* = 3.44 $\times \;10^{-8}$), GS 8 (*p* = 2.01 $\times \;10^{-4}$), GS 9 (*p* = 9.43 $\times \;10^{-4}$) and GS 7 versus GS 9 (*p* = 2.66 $\times \;10^{-3}$) (Figure 5H). When examining KRT8 expression by nodal status, a significant difference was found between normal tissues and N0 status (*p* = 9.41 $\times \;10^{-7}$) and normal versus N1 status (*p* = 9.95 $\times \;10^{-4}$). Finally, like CPM, KRT8 expression correlated with immune cell infiltration, specifically with B cells, CD4+ T cells, CD8+ T cells, macrophages, neutrophils, and dendritic cells (Figure 5I).

### 3.6. TCGA Bioinformatic Analysis: LRPCa vs. HRPCa

**Figure 6:**
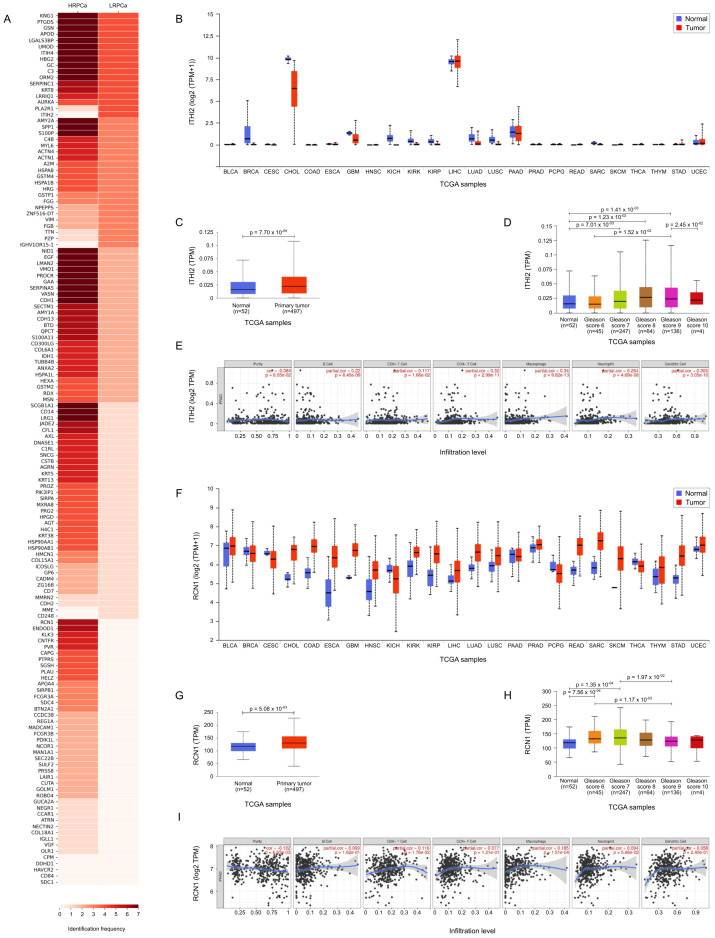
TCGA bioinformatic analysis. (**A**) Heatmap illustrating the frequency of protein expression in urine samples from prostate cancer-affected patients classified LRPCa and HRPCa. (**B**) TCGA expression data for Inter-alpha-trypsin inhibitor heavy chain H2 (ITIH2) across a pan-cancer dataset, comparing expression levels in tumor tissues (red) versus their normal counterparts (blue). (**C**) ITIH2 expression in normal vs. primary tumor. (**D**) ITIH2 expression in normal versus PCa tissues with different Gleason scores (GS). (**E**) TIMER analysis correlating ITIH2 expression with immune cell infiltration. (**F**) TCGA expression data for Reticulocalbin-1 (RCN1) across a pan-cancer dataset, comparing expression levels in tumor tissues (red) versus their normal counterparts (blue); Wilcoxon test *p* ≤ 0.05. (**G**) RCN1 expression in normal vs. primary tumor. (**H**) RCN1 expression in normal versus PCa tissues with different Gleason score (GS); Wilcoxon test *p* ≤ 0.05. (**I**) TIMER analysis correlating RCN1 expression with immune cell infiltration.

Following a comparison of protein expression frequency in PCa samples classified as LRPCa versus HRPCa, we observed that ITIH2 was present in all LRPCa (4 out of 4) but absent in HRPCa samples (*p* = 0.034), while RCN1 was exclusively expressed in HRPCa (6 out of 7) and not in LRPCa samples (*p* = 0.034). These findings, illustrated in Figure 6A and summarized in Table 2, suggest ITIH2 and RCN1 as potential distinguishing biomarkers for PCa risk stratification.

Figure 6B,F show the TCGA expression data for ITIH2 and RCN1, respectively, across a pan-cancer dataset, comparing expression levels in tumor tissues versus their normal counterparts. This analysis highlights distinct expression patterns for ITIH2 and RCN1, suggesting their potential roles as biomarkers across various cancers. In PCa patients specifically, expression differences for both proteins were statistically significant (*p* = 7.70 $\times \;10^{-5}$ for ITIH2 and *p* = 5.08 $\times \;10^{-3}$ for RCN1, shown in Figure 6C and Figure 6G, respectively). Further analysis of ITIH2 expression in normal versus PCa tissues revealed statistically significant differences across several Gleason scores, including normal prostate tissue versus GS 7 (*p* = 7.01 $\times \;10^{-3}$), GS 8 (*p* = 1.23 $\times \;10^{-2}$), GS 9 (*p* = 1.41 $\times \;10^{-3}$), GS 6 versus GS 9 (*p* = 1.52 $\times \;10^{-2}$) and GS 9 versus GS 10 (*p* = 2.45 $\times \;10^{-2}$) (Figure 6D). When examining ITIH2 expression by nodal status, a significant difference was found between normal tissues and N0 status (*p* = 5.22 $\times \;10^{-4}$), and normal tissue versus N1 status (*p* = 9.63 $\times \;10^{-3}$). Additionally, TIMER analysis indicated that ITIH2 expression is correlated with immune cell infiltration, specifically with B cells, CD4+ T cells, CD8+ T cells, macrophages, neutrophils, and dendritic cells (Figure 6E), suggesting a potential role for ITIH2 in the tumor microenvironment and immune response. Also RCN1 expression in normal versus PCa tissues revealed statistically significant differences across several Gleason scores, including normal prostate tissue versus GS 6 (*p* = 7.56 $\times \;10^{-4}$) and GS 7 (*p* = 1.35 $\times \;10^{-4}$), GS 6 versus GS 9 (*p* = 1.17 $\times \;10^{-2}$) and GS 7 versus GS 9 (*p* = 1.97 $\times \;10^{-3}$) (Figure 6H). When examining RCN1 expression by nodal status, a significant difference was found between normal tissues and N0 status (*p* = 1.29 $\times \;10^{-3}$), and normal tissue versus N1 status (*p* = 4.46 $\times \;10^{-2}$). Finally, like ITHI2, TIMER analysis indicated that RCN1 expression is correlated with immune cell infiltration, specifically with B cells, CD4+ T cells, CD8+ T cells, macrophages, neutrophils, and dendritic cells (Figure 6I).

## 4. Discussion

The analysis of the urinary proteome from HD and PCa patients enabled the identification of proteins that may play a role in the pathogenesis of this tumor, and that could be further investigated for diagnostic purposes. Many of them have already been mentioned in studies concerning PCa. For instance, YWHAB protein was included in a panel of molecules that contribute to the development of androgen-dependent (LNCaP) and androgen-independent (PC-3) prostate adenocarcinoma cell lines [60]. SERPINC1, although not yet correlated with PCa, was found differentially expressed in both PCa and benign tissues [51]. While HSP70 [62] and HSP90 [63] protein families overexpression seems to promote resistance, invasion and metastasis in PCa. Other proteins were instead correlated to PCa through transcriptomic studies. This is the case of KIF23, which was a hub gene in M2-tumor-associated macrophages, and it showed a higher expression level in tumor tissues [61], similarly to YWHAQ [59] and AGRN [65]. AGRN was also proposed as a substrate of Kallikrein-related peptidase 14 (KLK14), a serine protease involved in prostate cancer (PCa) pathogenesis [64]. However, the CDC5L-AGRN circuit was proposed as crucial for the oncogenic role of NEAT1 in PCa cells [65]. Furthermore, AGRN overexpression was observed in other tumors, i.e., head and neck cancer [43], where it also played the role of a hub.

Although overall our results are consistent with other findings already reported in the literature, it is necessary to underline the different nature of the samples (biological fluid, tissues, cell lines), the different analytical methods (proteomics, transcriptomics) and their sensitivity. As well as, in some cases, the non-specificity for prostate cancer. On the other hand, however, this agreement strengthens the role of the selected proteins in the context of the mechanisms underlying tumor diseases, as well as the role they could play for diagnostic purposes. In this scenario, we put KRT8 under the magnifying light through TCGA analysis. It encodes a cell surface protein commonly observed in PCa and recently linked to the expression of the non-coding RNA LINC00624, which may play a role in promoting PCa progression [69]. Also, KRT8 promotes the progression of epithelial–mesenchymal transition and cell migration. In PCa, they are enhanced by the stability of KRT8, which is mediated through the acetylation of its mRNA by N-acetyltransferase 10 (NAT10) [70]. Of note, the keratin genes, including KRT8 and KRT19, were found to be enriched in PCa cell lines from African American men compared to those from European American men, representing biomarkers with potential benefit for these patients [67]; a fact that highlights the difficulty of finding one or a few markers that can work for all cases and the need to analyze as many cases as possible to identify a multi-biomarker panel. Moreover, their role in the context of PCa was remarked by their topological relevance as hubs/bottlenecks, fitting with what was already reported in other works based on transcriptomics and PPI network analysis [66,71].

In terms of diagnostic potential, our study allowed the identification of new potential biomarkers present in the urine of PCa patients. On the other hand, it also highlighted proteins, including CPM and EGF, that characterize HD urine while their abundance decreased in disease states. CPM plays a key role in several physiological processes, including blood coagulation and fibrinolysis, inflammation, digestion, and the processing of pro-hormones and neuropeptides. Of particular interest is CPM’s constitutive expression in an active form on the surface of specialized cells and tissues throughout the body, suggesting its potential significance as a health-associated marker [72]. Notably, both CPM and EGF have been correlated in urine where CPM metabolize EGF to produce des-Arg53-EGF [54]. In lung adenocarcinomas, des-Arg53-EGF binds to the EGF receptor (EGFR), and the tissue expression of CPM has been negatively correlated with disease survival [73]. On the contrary, in colorectal cancer, CPM expression was positively correlated with overall survival and negatively correlated with recurrence, lymph node invasion, and N stage [74]. Although, in addition to the studies already cited, there are no other works that shed light on the significance of CPM in urine, its correlation with EGF [54] could suggest its presence as a factor of normality. In fact, while EGF is nonexistent or hardly detectable in plasma, it is present in normal people’s urine. Its decrease may indicate tubular atrophy and interstitial fibrosis, which could be associated with the risk and diagnosis of chronic kidney disease [53].

We also focused our efforts on selecting proteins that characterize patients at different risk levels. Although aware of the need to increase the sample size, we specifically detected ITIH2 in the urine of LRPCa patients. By excluding kidney and bladder cancer interference, it has been proposed as a urinary biomarker for PCa also by Lima et al. [47]. Supporting our findings, they observed a similar trend where ITIH2 expression decreased in tumor tissues, with distinct differences between adjacent normal tissue, LRPCa, and HRPCa samples. In addition to contributing to extracellular matrix stability by covalent linking to hyaluronic acid, ITIH proteins were shown to play an important role in inflammation and carcinogenesis [75]. The recruitment of inflammatory and immunocompetent cells has been related to complement activation [76]. Quantitatively, we found that some components of complement and coagulation cascades are characteristic of LRPCa urine. The modulation of the complement system during the early stages of PCa may play a significant role in the tumor microenvironment and immune evasion. Its involvement was documented in both PCa and prostate benign tissues, as well as in groups with varying metastatic tendencies [51]. Furthermore, it emerged as a proteomic signature of PCa in peripheral blood [57].

The strongest biomarker candidates for HRPCa included RCN1, CFL1 and HELZ. The downregulation of HELZ was correlated with a reduced translational initiation and a reduction in cell proliferation, but no studies have yet connected it to PCa or urine [77]. Differently, the cytoskeletal protein CFL1 was identified in both benign prostatic hyperplasia and PCa tissues as a histopathological biomarker candidate to avoid the misdiagnosis [78,79]. However, its identification in urine has not been shown to improve the early diagnosis of PCa [58]. RCN1 is instead a calcium-binding protein located in the lumen of the ER, while in human endothelial and prostate cancer cell lines, it is plasma membrane-localized [80]. RCN1 was linked to cancer progression and its downregulation significantly suppresses PCa cell viability and arrests the cell cycles of DU145 and LNCaP cells, highlighting its potential as a therapeutic target in cancer treatment [81]. Also in this case, unfortunately, no evidence is available about its presence in urine samples.

To shed light on the dysregulated molecular processes in PCa patients, our study highlighted the topological relevance of the identified proteins within a network structure, positioning urine as a mirror of alterations in prostate cancer tissue. Overall, a significant number of hubs/bottlenecks were associated with the cell cycle in both LRPCa and HRPCa patients, while others were related to proteolysis, immune response, and complement and coagulation cascades. In addition to microtubule-related proteins, such as KIF23 [61] and TUBB4B [51], which could easily be related to proliferative processes, the LRPCa hubs/bottlenecks included proteins from the HSP70 family, including HSPA1L and HSPA8. Conversely, the HRPCa group was characterized by the presence of HSP90 family components as hubs. In both cases, their increased expression in PCa was linked to promoting tumor cell survival and invasion making them potential therapeutic targets [62,63]. As for functional modules that are differentially abundant, glutathione metabolism emerged as a hallmark of HRPCa. By comparing the transcriptome of PCa and adjacent normal tissues, other studies identified hubs falling in this pathway [82]. Recently, the diagnostic potential of GSTP1 methylation in serum has been explored [83]. However, both GSTP1 and GSTM2, which showed increased abundance in HRPCa, did not demonstrate significant correlations with PCa progression in urine samples [84]. Despite this, oxidative stress has been implicated in the initiation and progression of prostate carcinogenesis through various mechanisms [85].

Finally, our attention was drawn by the presence of two heparan sulfate proteoglycans, SDC1 and SDC4, as HD hubs/bottlenecks. SDC1 plays a critical role in cell adhesion and maintaining epithelial integrity. By investigating prostate epithelial cells and tissues, Farfan et al. identified ZEB1 as a key repressor of SDC1 during PCa progression, highlighting that SDC1 expression is inversely related to PCa aggressiveness [68]. However, another study provided conflicting results, associating SDC1 with more aggressive tumors and worse prognosis, while SDC4 overexpression with better prognosis [86]. On the contrary, we found the identification of proteins that may function as tumor suppressors and angiogenesis inhibitors interesting in HD. Among them, we focused on Cell adhesion molecule 4 (CADM4) and Roundabout homolog 4 (ROBO4). CADM4 was described as a tumor suppressor gene in multiple cancers, including PCa [87], while Roundabout homolog 4 (ROBO4) is a transmembrane receptor specifically expressed in endothelial cells. It stabilizes the vascular network by inhibiting pathological angiogenesis and endothelial hyperpermeability [55]. In the context of PCa, it was found expressed in cancer epithelial cells and in the surrounding tumor stroma. Of note, higher histological tumor grade was correlated with the increased expression of ROBO4, while controversial patients with high ROBO4 showed lower recurrence, suggesting its protective role [88].

## 5. Conclusions

Our study aimed to identify proteins and biological processes dysregulated in PCa patients, using urine as a mirror of the changes occurring in prostate tumor tissue [47]. In this scenario, the combination of proteomic and network analysis allowed the discovery of new putative biomarkers that could contribute to improving the performances of the available diagnostic test [89]. We are well aware that the number of samples taken into consideration should be greater. For this reason, our study did not aim to discover a universal set of discriminating proteins but rather to contribute to the knowledge already available for the implementation of a panel of multiple markers. However, the integration of our results with the TCGA database and bioinformatic tools has preliminarily validated our findings across a broader in silico population. It highlights the potential of our approach to shed light on proteins that, in addition to putative biomarkers, may play a key role in the processes impaired by PCa. Indeed, the good overlap of our results with those already reported in the literature increases the confidence that the selected proteins contribute to the mechanisms underlying this disease.

Finally, in the landscape of issues that are being discussed, we must consider that, in our study, the average age of HD subjects is significantly different from that of PCa patients, with them being older. While aging is a known risk factor for both PCa incidence and mortality, there has been an increase in incidence among younger men since the late 1980s, with notably lower survival rates compared to older men [90]. However, the likelihood of being affected by indolent PCa is higher in older individuals than in younger ones. Therefore, although aging cannot be completely excluded as a potential confounder factor influencing urine proteomics [91], it is important for the HC subjects to be younger than the PCa patients enrolled in the study. Moreover, it should be emphasized that, if our findings are validated in further studies involving a larger population cohort and age-matched case-controls, they could complement, but not replace, current diagnostic strategies such as PSA serum levels, imaging, and biopsy sampling.

All these factors contribute to painting a complex and heterogeneous picture, where ethnic groups and geographic regions also contribute to different molecular, pathological and disease outcome subtypes [67]. This is a further good reason to improve the knowledge of PCa with as many studies as possible that can cover the panel of variables that characterize it.

## Figures and Tables

**Table 1:** Proteins significantly discriminating urine of HD subjects (*n* = 5) from those of patients diagnosed with PCa (LRPCa and HRPCa, *n* = 11); Wilcoxon test (*p* ≤ 0.05). Carboxypeptidase M (CPM) and Keratin, type II cytoskeletal 8 (KRT8) were those most abundant in HD and PCa patients, respectively.

UNIPROT ID	Protein Name	Gene Name	HD	PCa	*p*-Value
F8W111	Carboxypeptidase M	*CPM*	5 (100%)	0 (0%)	0.00063
O75882	Attractin	*ATRN*	5 (100%)	1 (9%)	0.0034
A0A3B3ISU3	Low affinity immunoglobulin gamma Fc region receptor III-B	*FCGR3B*	5 (100%)	2 (18%)	0.012
Q16651	Prostasin	*PRSS8*	5 (100%)	2 (18%)	0.012
Q8WZ75	Roundabout homolog 4	*ROBO4*	5 (100%)	2 (18%)	0.012
Q9H8L6	Multimerin-2	*MMRN2*	5 (100%)	2 (18%)	0.012
P19022	Cadherin-2	*CDH2*	5 (100%)	2 (18%)	0.012
P05787	Keratin, type II cytoskeletal 8	*KRT8*	0 (0%)	10 (91%)	0.0034
P25815	Protein S100-P	*S100P*	1 (20%)	10 (91%)	0.024
A8MY60	Leucine-rich repeat and IQ domain-containing protein 1	*LRRIQ1*	0 (0%)	9 (82%)	0.012

**Table 2:** Proteins significantly discriminating urine of LRPCa patients (*n* = 4) from those of HRPCa patients (*n* = 7); Wilcoxon test (*p* ≤ 0.05). Inter-alpha-trypsin inhibitor heavy chain H2 (ITIH2) and Reticulocalbin-1 (RCN1) were those most abundant in LRPCa and HRPCa, respectively.

UNIPROT ID	Protein Name	Gene Name	LRPCa	HRPCa	*p*-Value
P19823	Inter-alpha-trypsin inhibitor heavy chain H2	*ITIH2*	4 (100%)	0 (0%)	0.034
Q13018	Secretory phospholipase A2 receptor	*PLA2R1*	4 (100%)	1 (14%)	0.034
Q15293	Reticulocalbin-1	*RCN1*	0 (0%)	6 (86%)	0.034
O94919	Endonuclease domain-containing 1 protein	*ENDOD1*	0 (0%)	6 (86%)	0.034
P11684	Uteroglobin	*SCGB1A1*	1 (25%)	7 (100%)	0.047
P08571	Monocyte differentiation antigen CD14	*CD14*	1 (25%)	7 (100%)	0.047
P02750	Leucine-rich alpha-2-glycoprotein	*LRG1*	1 (25%)	7 (100%)	0.047

## Data Availability

Proteomics raw data used in this study have been deposited in MassIVE database ftp://MSV000096381@massive.ucsd.edu.

## Supplementary Materials

The following supporting information can be downloaded at: https://www.mdpi.com/article/10.3390/biology14030256/s1, Table S1: List of proteins identified; Table S2: List of differentially abundant proteins; Table S3: Differentially enriched GO terms and pathways; Table S4: Hubs and bottlenecks.

## Abbreviations

The following abbreviations are used in this manuscript:

PSA	Prostate-Specific Antigen
PCa	Prostate Cancer
TCGA	The Cancer Genome Atlas
DRE	Digital Rectal Examination
TRUS	Transrectal Ultrasound
MRI	Magnetic Resonance Imaging
GS	Gleason Score
mpMRI	multi-parametric Magnetic Resonance Imaging
CT	Computed Tomography
PET	Positron Emission Tomography
ISUP	International Society of Urological Pathology
NCCN	National Comprehensive Cancer Network
CAPRA	Cancer of the Prostate Risk Assessment
AI	Artificial Intelligence
PPI	Protein-Protein Interaction
HD	Healthy Donors
LRPCa	Low-risk PCa patients
HRPCa	High-risk PCa patients
TFA	Trifluoroacetic Acid
LC	Liquid Chromatography
MS	Mass Spectrometry
FDR	False Discovery Rates
BP	Biological Process
MF	Molecular Function
CC	Cellular Component
LDA	Linear Discriminant Analysis
PSM	Peptide Spectrum Match
DAPs	Differentially Abundant Proteins
IF	Identification Frequency
DAve	Differential Average
PCA	Principal Component Analysis
nLC-hrMS/MS	Nano-Liquid Chromatography-Mass Spectrometry/
	High Resolution Mass Spectrometry
PC1	Principal Component1
PC2	Principal Component2
HSPs	Heat Shock Proteins (HSPs)
BPH	Benign Prostatic Hyperplasia
